# Transcatheter Aortic Valve Implantation in a Ross Procedure Patient With Severe Autograft Regurgitation: A Case Report

**DOI:** 10.1002/ccd.70019

**Published:** 2025-07-22

**Authors:** Anindya Mukherjee, Nikhil Joshi, Stephanie Curtis, Mark Turner

**Affiliations:** ^1^ Scarborough General Hospital Scarborough UK; ^2^ University of Bristol Bristol UK; ^3^ Bristol Heart Institute Bristol UK

**Keywords:** autograft regurgitation, case report, ross procedure, TAVI

## Abstract

Surgical aortic valve replacement (AVR) is the standard treatment for symptomatic severe neo‐aortic regurgitation in Ross patients. The only previous report of a transcatheter aortic valve implantation (TAVI) in a Ross patient required valve‐in‐valve rescue. Thus, this is the first uncomplicated TAVI procedure in a Ross patient.

**Abbreviations**
AR: aortic regurgitation
AS: aortic stenosis
AVR: aortic valve replacement
BMI: body mass index
CE: European Conformity
CMR: cardiac magnetic resonance
CT: computed tomography
ECG: electrocardiogram
LVOT: left ventricular outflow tract
PPVI: percutaneous pulmonary valve implantation
PVR: pulmonary valve replacement
TAVI: transcatheter aortic valve implantation
TEE: trans‐esophageal echocardiography
THV: trans‐catheter heart valve
TTE: trans‐thoracic echocardiography

## 1 Introduction

The Ross procedure is an excellent alternative to prosthetic aortic valve replacement (AVR) in some patients, but autograft failure, with dilation and regurgitation, requires reintervention in approximately 10% of patients at 15 years [1, 2]. Surgical AVR is the standard treatment for symptomatic severe neo‐aortic regurgitation in Ross patients. The only previous report of a transcatheter aortic valve implantation (TAVI) in a Ross patient required valve‐in‐valve rescue [3]. Thus, this is the first report of an uncomplicated TAVI procedure in a Ross patient.

## 2 History of Presentation

A 45‐year female who had undergone a Ross procedure for congenital aortic stenosis 25 years earlier presented with progressive symptoms over the prior year consisting of breathlessness, exercise intolerance, orthopnea, postural dizziness, palpitations, and lower extremity edema. She denied chest pain. She was treated with metformin for type II diabetes and had a BMI of 43 kg/m^2^. Details of the Ross procedure could not be obtained.

## 3 Differential Diagnosis

As there was both leaflet thickening and regurgitation across the 26‐year‐old autograft with no evidence of endocarditis, leaflet degeneration seemed the most likely pathology.

## 4 Investigations

**Figure 1:**
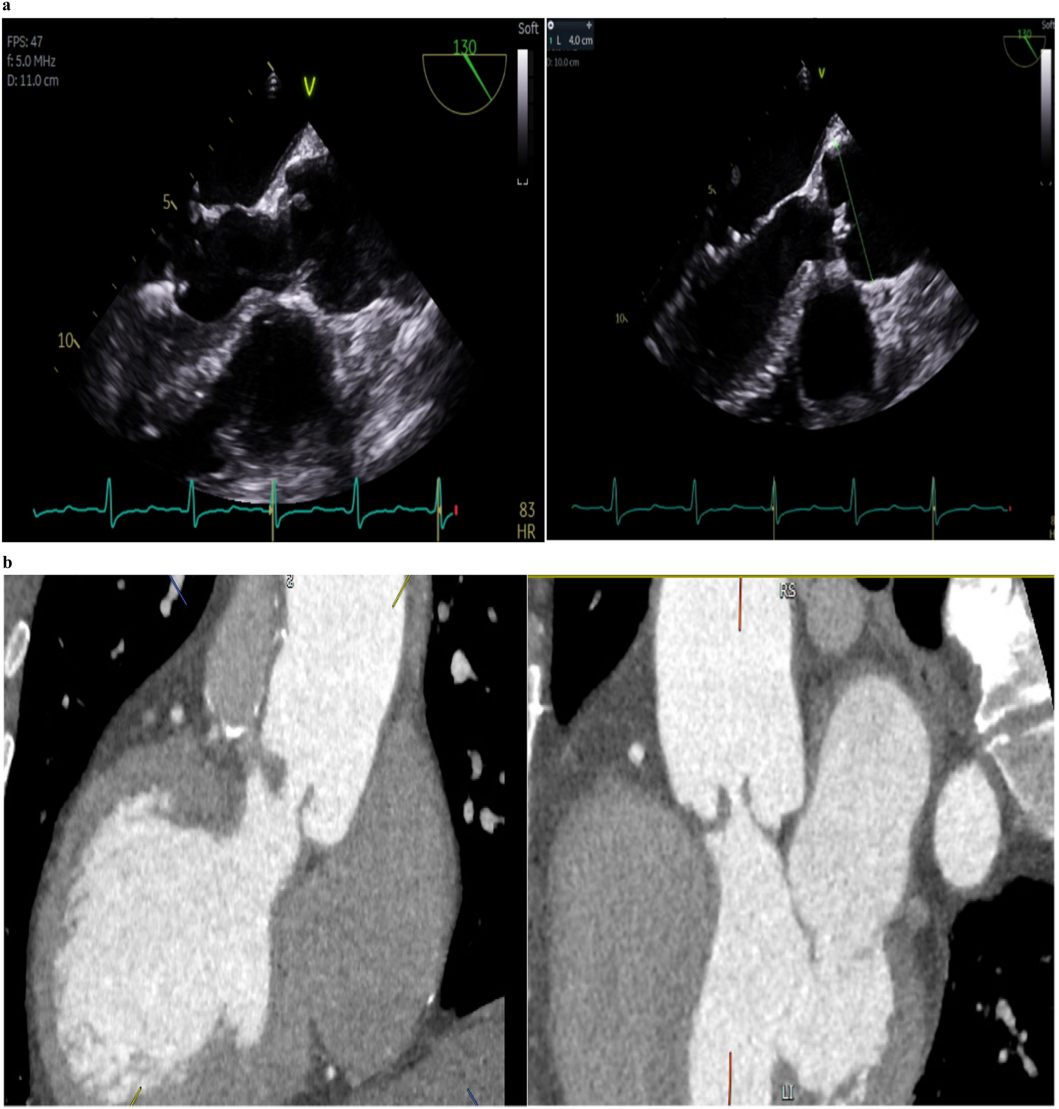
Pre‐procedural imaging. (a) TEE demonstrating the pathology. Severe neo‐aortic regurgitation due to fixed anterior leaflets and dilatation of neo‐aortic root causing central coaptation failure. (b) Pre‐procedural CT. Demonstration of the LVOT anatomy in the Ross patient with fixed anterior leaflets, and dilated neo‐aortic root causing central coaptation failure. The elongated LVOT formed by anastomosis between autograft and native LVOT forms the landing zone of the future TAVI valve. [Color figure can be viewed at wileyonlinelibrary.com]

Trans‐thoracic (TTE) and transoesophageal echocardiography (TEE) revealed severe neo‐aortic regurgitation and mild neo‐aortic stenosis (Figure 1a, Video S1). There was mild pulmonary homograft stenosis and moderate regurgitation (Video S2). Maximum velocity across the aortic valve was 3.5 m/s, mean pressure gradient was 29 mmHg, AR pressure half time was 254 ms. Diastolic flow reversal was noted in supra‐sternal view with velocity time integral of 27 cm.

Computed tomography (CT) aortogram revealed normal coronaries with right coronary height 14 mm, left main coronary height 16 mm, aortic annulus 29 × 25 mm (area 563 mm^2^ measured in end diastole), sinus of Valsalva 38 mm, sinotubular junction 40 × 42 mm and proximal ascending thoracic aorta of 45 mm (Figure 1b). The femoral artery diameters were 7 mm on the right and 6 mm on the left.

## 5 Management (Medical/Interventions)

The patient was on optimal medical treatment including beta‐blockers and diuretics. She was discussed in the adult congenital heart disease multidisciplinary team meeting, and the options discussed were: TAVI followed by—percutaneous pulmonary valve implantation (PPVI) later or surgical mechanical AVR along with pulmonary valve replacement (PVR). The team felt TAVI was the best option for her at that time as (i) the ascending aorta did not require replacement, (ii) the pulmonary homograft disease did not warrant intervention, (iii) her anatomy was suitable for TAVI with coronary heights > 7 mm, and sufficiently large aortic sinuses, (iv) diabetes with increased BMI and (v) surgical option with a longer hospital stay followed by home‐based convalescence would have an impact on her ability to care for her daughter.

The technical challenge for TAVI was that there was aortic regurgitation and very little calcium to anchor the valve. While planning the procedure, we thought of the strategy to deal with potential complications. The autograft leaflets were thickened; there was some AS and the anastomosis between the autograft and native LVOT provided a relatively rigid landing zone‐ these three features along with rapid cardiac pacing while deployment would stabilize and hold the valve in place. We used a Medtronic Evolut R which allowed us to recapture the valve when it popped up into the aorta during initial deployment at a standard depth and was easy to reposition lower in the LVOT at the anastomosis between native LVOT and autograft (Video S3), guided mainly by TEE as angiography was unclear because of the regurgitation. Rapid pacing was done during deployment (Video S3). The surgical team was available, and the procedure was performed in a hybrid catheterization laboratory. We were ready to go onto femoral access cardiopulmonary bypass if needed. The plan was made to implant a 29 mm valve, under TEE guidance after thorough discussion with the patient and her written informed consent. We measured the narrow part of LVOT which averaged at 25 mm; therefore, we chose this particular valve as we knew the anastomotic site was relatively non‐distensible.

**Figure 2:**
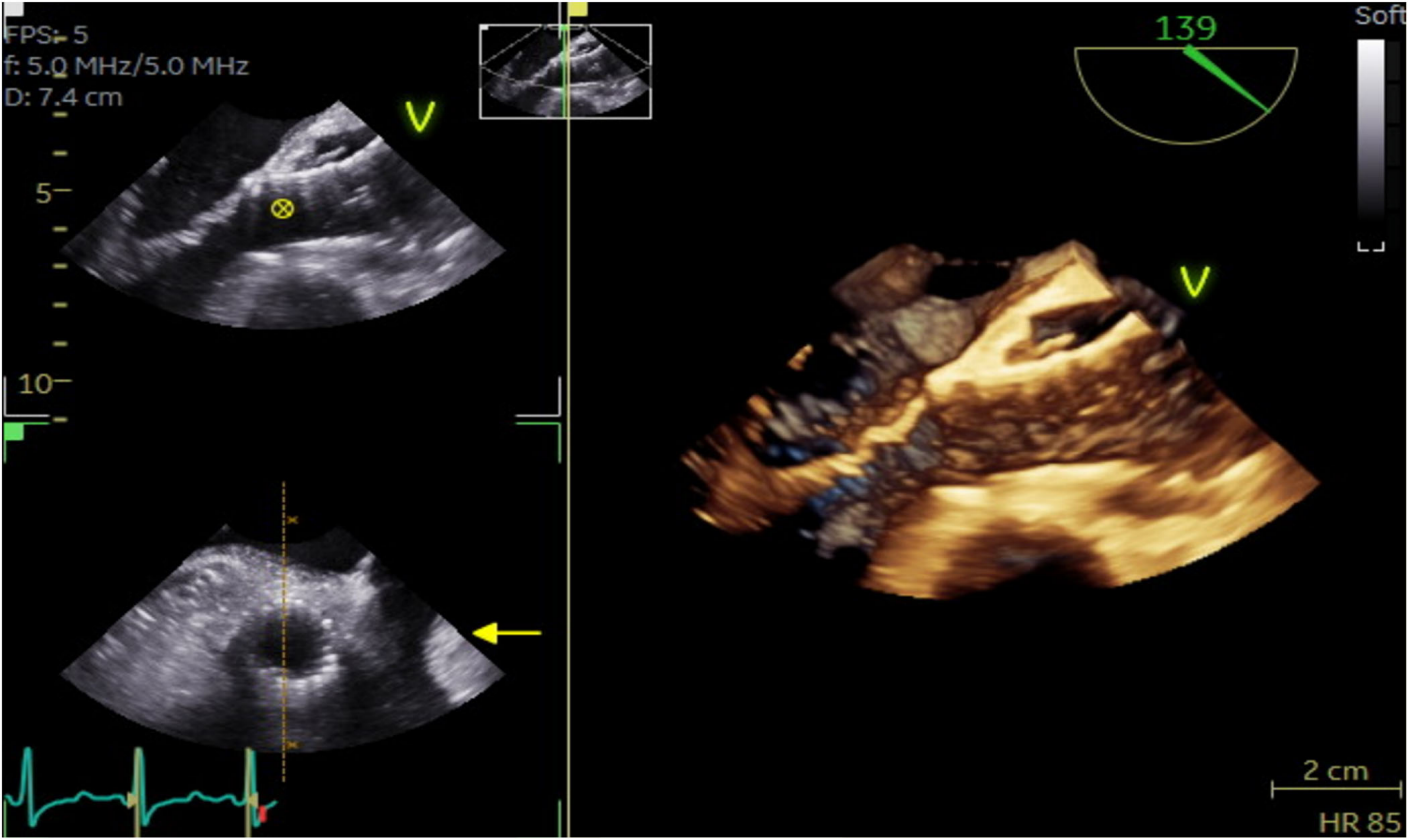
Post‐procedural TEE. Well opposed TAVI valve with no residual stenosis or regurgitation. [Color figure can be viewed at wileyonlinelibrary.com]

The post‐implant hemodynamics showed the AR index to be 50 (a good result would be >25). Post‐intervention TEE showed a good result (Figure 2, Video S4) and TTE showed peak velocity of 2.2 m/s across the aortic valve with a mean gradient of 10 mmHg and no regurgitation (Video S4).

## 6 Discussion

Surgical AVR is the standard treatment for symptomatic severe AR but according to recent guidelines, TAVI may be considered for selected patients in experienced centers [4]. The increased risk of transcatheter heart valve (THV) embolization, migration or paravalvular leak in the absence of aortic valve calcification makes TAVI challenging in severe pure AR [5]. JenaValve has received a CE mark for the treatment of AR, AS and the Acurate neo THV for pure non‐calcified AR has demonstrated safety and efficacy in a recent case series of selected patients [6, 7].

**Figure 3:**
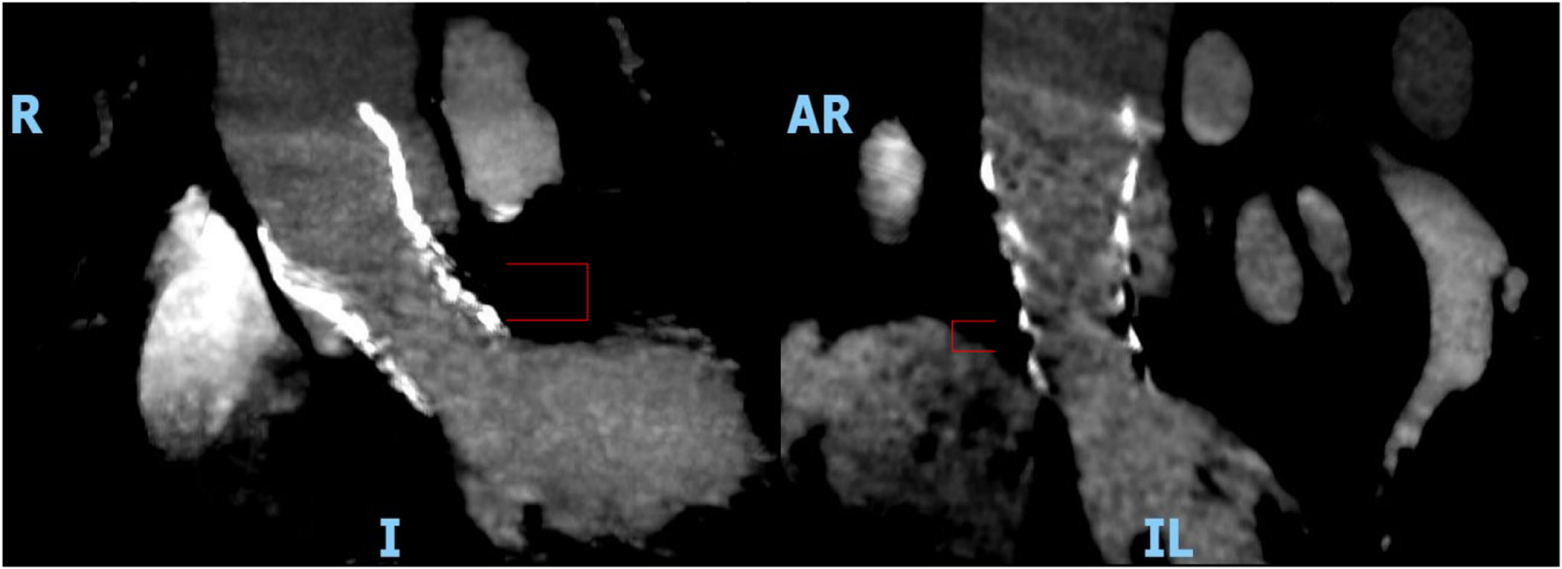
Post‐procedure CT image. Subannular positioning of the TAVI valve, anastomosis between autograft and native LVOT is the landing zone (annotated by the red third bracket). [Color figure can be viewed at wileyonlinelibrary.com]

The first TAVI for severe AR in a Ross autograft was performed in a young woman 10 years after the Ross operation, as the patient was deemed inoperable. A 29 mm CoreValve (Medtronic, Minneapolis, MN, USA) was used, which was not recapturable and repositionable. Residual severe paravalvular leak necessitated deployment of a second prosthesis “valve‐in‐valve‐in‐valve”. The leak was thought to be caused by the peculiar post‐surgical anatomy [3]. This led to the thought that the residual leak could have been prevented by implanting the first prosthesis much lower than the autograft valve level where the autograft is sutured to the LVOT. In our case we planned to implant the valve at a much lower level than usual to seal in the autograft to the left ventricular outflow tract (LVOT) anastomosis. With this approach, a single valve was successful, although one recapture and repositioning was needed (Figure 3, Video S3, Video S4). As demonstrated by the PANTHEON International Project, despite the advancements of platforms and techniques, TAVI for severe native aortic valve regurgitation remains challenging with risk of valve embolization and migration [8]. The post‐implant AR index was 50 in this procedure. The study by Sinning et al. demonstrated that patients with AR index < 25 had a significantly increased 1‐year mortality risk compared to those with AR index ≥ 25 and the mean AR index in patients without periprosthetic AR was 32 ± 10 [9]. We had previously treated an aortic homograft with a similar implant technique and found that the TAVI valve worked well when implanted at the homograft‐LVOT anastomosis level [2].

## 7 Follow‐up

The patient symptomatically improved after the TAVI and has no cardiac symptoms 5 years after the procedure. She no longer requires diuretics and TTE performed 4 years after TAVI shows no AR and good left ventricular function (video S5). The cardiac magnetic resonance imaging performed at 5 years showed good bi‐ventricular function. The pulmonary valve function has not deteriorated and has only moderate pulmonary regurgitation.

## 8 Conclusion(s)

The excellent haemodynamic and clinical results in our case shows the feasibility of TAVI for severe autograft regurgitation after a Ross operation where a landing zone for the TAVI valve can be identified. Individual anatomic and hemodynamic features should be carefully considered before undertaking TAVI in a patient with AR after a Ross procedure.

## Conflicts of Interest

Mark Turner is a consultant and proctor for Edwards, Medtronic, Abbott, Occlutech and Gore. The other authors declare no conflicts of interest.

## Supporting information


**Video 1: Pre procedure neo‐aortic valve in TTE (left panel) and TEE (right panel).** Severe neo‐aortic regurgitation due to fixed anterior leaflets and dilatation of neo‐aortic root causing central coaptation failure.

**Video 2: Pulmonary valve assessments.** Mild pulmonary homograft stenosis and moderate regurgitation.

**Video 3: Fluoroscopic video images of the actual deployment of the valve.** 1^st^ pane showing initial deployment and popping up of TAVI valve into the aorta. 2^nd^ pane showing at the second attempt, the valve was deployed to a lower position, at the anastomosis between native LVOT and autograft. 3^rd^ pane showing final deployment position in relation to the neoaortic valve.

**Video 4: Post procedure aortic valve in TTE (left panel) and TEE (right panel).** Post‐intervention TEE showed a good result and TTE showed peak velocity of 2.2 m/second across the aortic valve with a mean gradient of 10 mm Hg and no regurgitation.

**Video 5: 4 years post procedure TTE.** Stable TAVI valve and good left ventricular function.
