# Supplementary material for: Transcatheter Aortic Valve Implantation in a Ross Procedure Patient With Severe Autograft Regurgitation: A Case Report
Source: Catheter Cardiovasc Interv. 2025 Jul 22;106(3):1946–50. doi: 10.1002/ccd.70019 (PMC12412343; doi:10.1002/ccd.70019)
Supplement: Supplementary file 3 — Video 3: Fluoroscopic video images of the actual deployment of the valve. 1st pane showing initial deployment and popping up of TAVI valve into the aorta. 2nd pane showing at the second attempt, the valve was deployed to a lower position, at the anastomosis between native LVOT and autograft. 3rd pane showing final deployment position in relation to the neoaortic valve. [file CCD-106-1946-s005.pptx]

## Slide 1
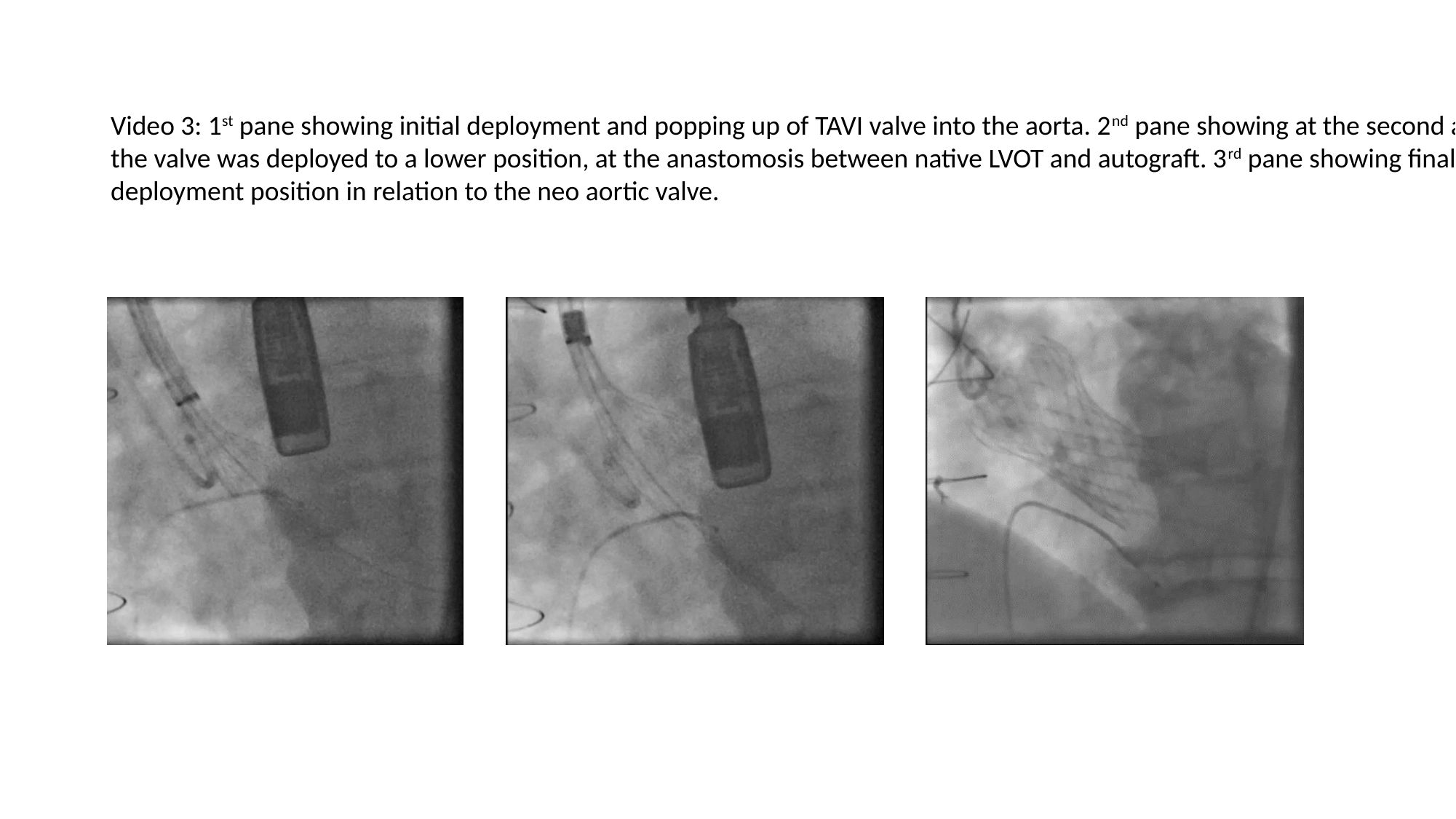

Video 3: 1st pane showing initial deployment and popping up of TAVI valve into the aorta. 2nd pane showing at the second attempt, the valve was deployed to a lower position, at the anastomosis between native LVOT and autograft. 3rd pane showing final deployment position in relation to the neo aortic valve.
